# Persistent Hiccups As Presenting Symptom of COVID-19: A Case of 64-Year-Old Male From International Medical Center, Jeddah, Saudi Arabia

**DOI:** 10.7759/cureus.20158

**Published:** 2021-12-04

**Authors:** Mohammed I Habadi, Nashaat Hamza, Tarig H Balla Abdalla, Afnan Al-Gedeei

**Affiliations:** 1 Family Medicine, University of Jeddah, Jeddah, SAU; 2 Infectious Disease, International Medical Center, Jeddah, SAU; 3 Internal Medicine, University of Toronto, Toronto, CAN; 4 Family Medicine, International Medical Center, Jeddah, SAU

**Keywords:** covid-19 hicupps, covid-19 presentation, hiccups, persistent hiccups, intractable hiccups

## Abstract

The possibilities of coronavirus disease 2019 (COVID-19) to present with atypical manifestations have reported. Information of COVID-19 atypical signs and symptoms is still emerging globally. One of these presentations is persistent hiccups. One of the hypotheses is that COVID-19 has been linked to several neurological manifestations and effects. Some observations noticed phrenic nerve paralysis after COVID-19 infection leading to pulmonary failure. We report one case of COVID-19-positive patient where he presented with persistent hiccups. Many predisposing factors might lead to the development of hiccups in COVID-19 infection such as a history of smoking, phrenic and vagus nerve damage or irritation, high inflammatory markers, lower lobe pneumonia, ground-glass-like appearance on x-rays. We hypothesize that hiccups are the first sign of serious deterioration of patients with COVID-19 and such patients are at high risk of developing kidney injury and intubation.

## Introduction

Singultus (hiccups) is a common anatomical and physiological response that is caused by irritation to the muscles of the diaphragm [1]. Hiccups are considered a sudden abnormal physiological behavior resulting in a contracture of the involuntary diaphragmatic and intercostal muscles; they are believed to be produced initially in the fetus to train the respiratory muscles in the uterus [1,2]. There has been speculation that some eating spells and disruption of the gastrointestinal tract may also produce them [2]. They are divided into acute hiccups, which are present in the first 48 hours, and persistent hiccups, which last for more than 48 hours [1]. Acute hiccups are usually self-limited and represent no clinical significance. However, persistent hiccups should elicit clinical significance and may have an underlying etiology [1]. Causes of hiccups may include cardiovascular (CVS) disorders, central nervous system (CNS) disorders, otolaryngology disorders, infectious disorders, intrathoracic disorders, gastrointestinal disorders, endocrine disorders, surgery, and/or drugs [3-5]. Hiccups mainly consist of three components that contribute to the mechanism of how they are produced. The first component is the afferent limb, which includes the vagus, phrenic and sympathetic nerves supplying the viscera [6]. The second component is the central processing unit, which involves the brain stem, medulla oblongata, temporal lobes, chemoreceptors, glossopharyngeal and phrenic nerve nuclei, and hypothalamus [1-6]. Moreover, the central neurotransmitters related to the central processing unit include gamma-aminobutyric acid (GABA), dopamine, and serotonin [7]. The last component is the efferent component, which includes the phrenic nerve supplying the diaphragm and the accessory nerves supplying the intercostal muscles [1]. The novel coronavirus disease 2019 (COVID-19) is an infectious disease that was declared a pandemic by the World Health Organization (WHO) [8]. Patients infected with the virus can present with an array of symptoms, such as fever, cough, fatigue, body aches, and sore throat [9]. Some patients still present with atypical manifestations and therefore can be important in the management of this disease [9]. Furthermore, various respiratory infectious diseases, such as pneumonia, bronchitis, pharyngitis, and laryngitis, have presented with hiccups [10,11]. In addition, excessive smoking, alcohol, and sudden changes in food and drink temperatures are known causes of hiccups [6]. Chlorpromazine is the sole medication used primarily to treat idiopathic persistent and prolonged hiccups. However, using it without exploring the main etiology is not recommended, as it may lead to missing a serious underlying disorder [6]. As a result, alternative remedies and a conservative approach are recommended initially while treating the underlying cause. In this case, we report a patient who presented with persistent hiccups as a symptom of COVID-19.

## Case presentation

**Figure 1:**
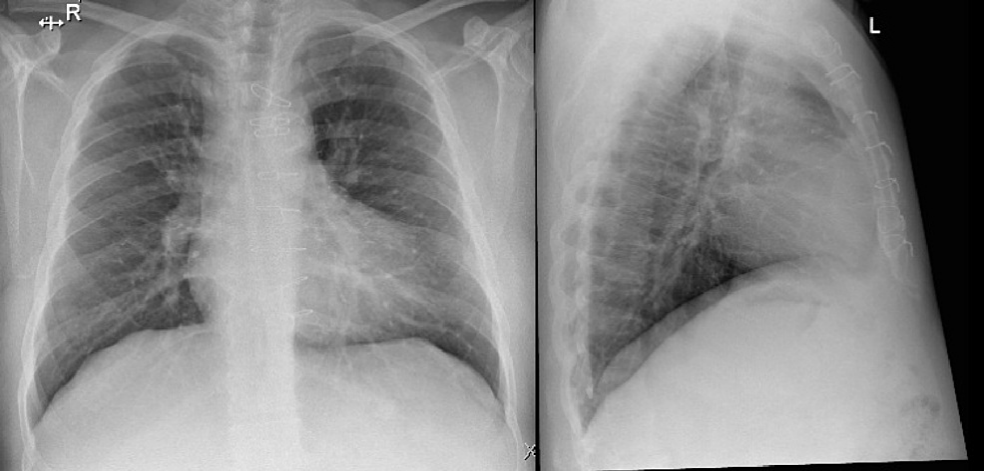
Chest x-ray of the patient at presentation

A 64-year-old male ex-smoker presented with type 2 diabetes mellitus (DM), hypertension, ischemic heart disease post-coronary artery bypass graft (CABG) 2017, dyslipidemia, a peripheral vascular disease with post right big toe amputation about four years ago, and erectile dysfunction. On August 8, 2020, the patient presented to the triage clinic of the International Medical Center, Jeddah, concerned about unprotected contact with another confirmed COVID-19 case. In addition, he reported a runny nose and a cough, with a feeling of fatigue as well as dizziness for three days prior to his presentation. Physical examinations, including vital signs and a pulmonary exam, were all normal. Accordingly, we ordered a nasopharyngeal swab and a chest x-ray (CXR), which were taken and sent to the same facility laboratory (Figure 1). The patient was advised to start on azithromycin 500 mg once every day for three days and to self-isolate until after the swab result.

In the patient follow-up the next day, the test result was positive. He was additionally prescribed cefuroxime (500 mg OD), dexamethasone (8 mg OD), and enoxaparin sodium (40 mg OD). He was advised to continue on home isolation. Five days after his first presentation, the patient had developed a sore throat and persistent hiccups despite improvement in his symptoms. Aside from his congested throat, the patient’s physical examination, including vital signs and lungs examination, was normal. Therefore, the patient was returned for self-isolation at home and advised to continue on the same treatment.

**Figure 2:**
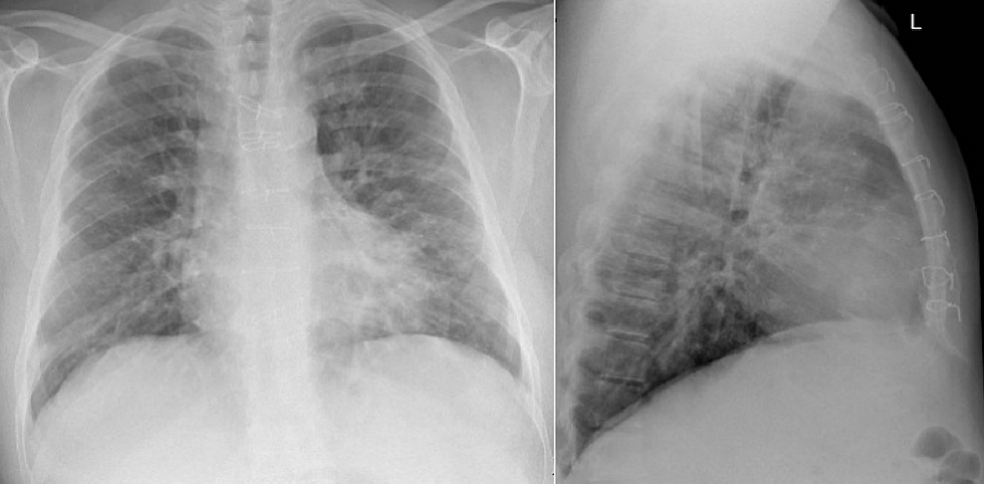
Chest x-ray of the patient on follow-up

On August 15, 2020, he presented again with shortness of breath and cough for a one-day duration. Vital signs were normal except mild tachypnea and the pulse oximetry revealed 94% oxygen saturation. A chest x-ray was ordered and the patient was admitted to an isolation room (Figure 2).

**Figure 3:**
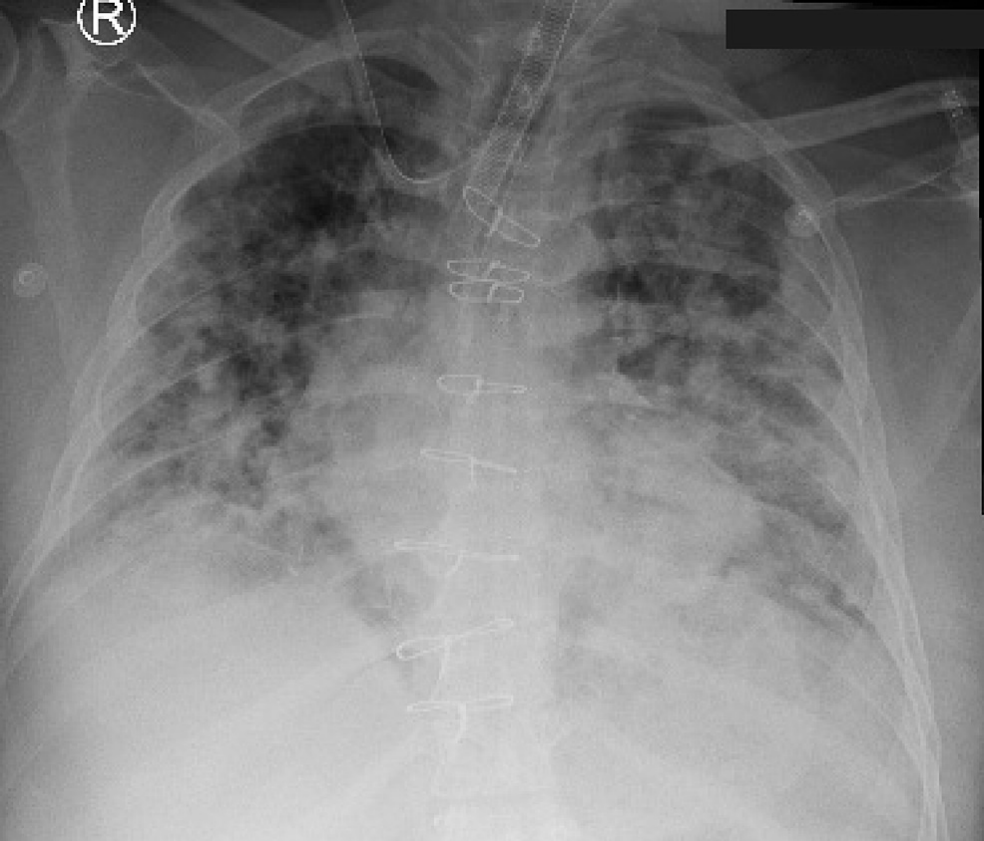
Chest x-ray of the patient during admission

On the second day of admission, the patient started on levofloxacin, but he developed an adverse reaction to the medication in the form of anaphylaxis (bronchospasm, tachycardia, respiratory distress). This attack was managed with epinephrine, hydrocortisone, and antihistamine, but the patient’s condition deteriorated. He was therefore intubated, mechanically ventilated, and shifted to the intensive care unit (ICU) for close monitoring. The patient’s course during admission in ICU fluctuated between minimal stability and frequent deteriorations (Figure 3).

**Figure 4:**
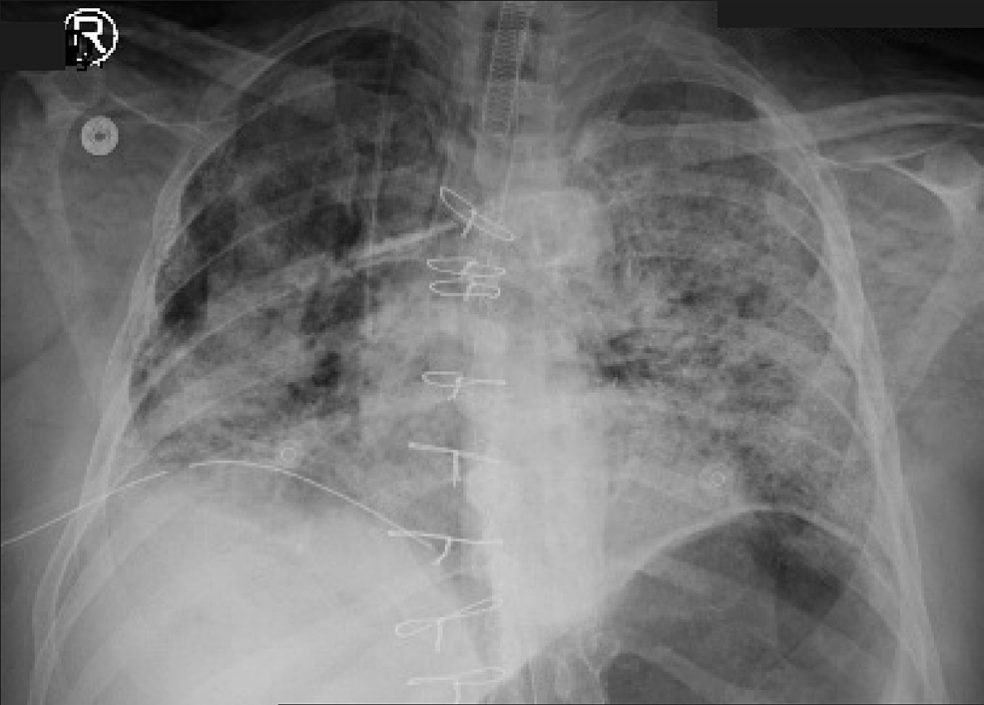
Chest x-ray of the patient during admission

The summary for the course during admission is shown in Table 1. On September 21, 2020, the patient developed severe respiratory failure and cardiac arrest in the form of Brady-asystole (Figure 4). This progressed despite aggressive resuscitation and he deceased.

**Table 1:** Summary of the course during admission COVID-19: coronavirus disease 2019; CRP: C-reactive protein; WBC: white blood cell; CXR: chest x-ray; GFR: glomerular filtration rate; ACLS: advanced cardiovascular life support; MCV: mean corpuscular volume; RDW: random distribution width; HCT: hematocrit; PRBCs: packed red blood cells; DC: discontinued; Echo: echocardiogram; GS: general surgery; ACC: active care continues; PCV: packed cell volume; UOP: urine output; Abx: antibiotic; CCRT: critical care response team

Date	Daily progress	Procedure done	Significant lab results
August 15, 2020	Patient has dyspnea and cough. Started on IV levofloxacin (750mg )	Admitted to the COVID-19 isolation room	Bilateral infiltrates in CXR; CRP:116.56
August 16, 2020	Patient is distressed was hypoxic (SPO_2_ 96% on room air); patient had an anaphylactic shock and ACLS protocol was applied.	Intubated and mechanically ventilated; central venous and arterial line inserted.	GFR: 57; creatinine serum: 1.27; urea serum: 31.45; CRP: 186.81; WBC: 9.79; neutrophils: 8.93; lymphocytes: 0.33
September 9, 2020	Patient is still sedated and ventilated on a ventilator with pressure control ventilation undigested parameters of hypercapnia and hypoxia	New left arterial line inserted; removal of the old femoral line; PRBCs transfused	Hemoglobin: 8.3; MCV: 83; RDW: 15; Hct: 24.4; platelet count: 106
September 10, 2020	Patient is still sedated and ventilated on a ventilator with pressure control ventilation undigested parameters of hypercapnia and hypoxia	On high FiO_2_ requirements with nitric oxide inhalation 10ppm; off vasopressors	
September 11, 2020	DC glycopyrrolate; resume heparin	Planned for ECHO next day	
September 12, 2020	Patient on full vent support; decreased the inspiratory pressure to reduce the leak through the right-sided pneumothorax	Right chest tube has been inserted by the GS team	ECHO: technically difficult study, poor echogenicity, limited echo views; overall normal left ventricle size, global and systolic function; mild concentric LV hypertrophy; normal right ventricle size and systolic function; no pericardial effusion; CXR: right chest wall surgical emphysema and rim pneumothorax
September 13, 2020	Chest drain is to be reviewed with the surgery team	Start on sildenafil; DC thiamine, ACC	
September 14, 2020	Patient sedated with propofol, fentanyl; still PCV; oliguria	Norepinephrine started; sildenafil stopped; IV Lasix started	
September 15, 2020	Patient is still sedated and ventilated with severe hypoxia and hypercapnia; no fever; UOP is anuric	On a small dose of norepinephrine; Lasix is running and not effective; antibiotic changed from ceftazidime to tazocin	
September 16, 2020	Wean no	Double NaHCO_3_ dose; adjust ABx dose; enema Movicol was added	
September 17, 2020	Still PCV; still CRRT on vasopressor	Off sedation but still not awake; Bactrim added	
September 18, 2020	Patient is still sedated and ventilated with severe hypoxia and hypercapnia; no fever; anuric	Antibiotics modified	
September 19, 2020	Patient is still on a small dose of norepinephrine; off sedation; still on pressure control ventilation with high FiO_2_ and respiratory acidosis; still on nitric oxide; right intercostals tube in situ	New COVID-19 assay is negative	
September 20, 2020	Patient in shock	Started on IV pressors; resumed CRRT	
September 21, 2020	Patient developed a severe respiratory failure and cardiac arrest in form of Brady-asystole: this has progressed despite aggressive resuscitation and deceased		Asystole on ECG

## Discussion

Information of COVID-19 atypical signs and symptoms is still emerging globally. Reporting new and unusual signs and symptoms is crucial in this pandemic and the management of those patients. Previous cases have been reported with the presentation of hiccups with COVID-19 [12-14]. Furthermore, smoking, pneumonia, and pulmonary embolism have been established in the literature as causes of persistent hiccups [15].

Our patient was an ex-smoker who contracted COVID-19 and presented with persistent hiccups of five days duration. He also developed ground-glass appearance pneumonia followed by pleural effusion. Typically, the reported cases of presenting hiccups with chest infection involve the lower lobes of the lung and types of atypical pneumonia [16]. Our patient developed pneumonia that involved the lower lobes. This pneumonia might have affected the phrenic nerve, which is one of the first components of the hiccup pathophysiology [6]. Moreover, COVID-19 has been linked to several neurological manifestations and effects. Maurier et al. observed a case of a non-smoker obese patient with phrenic nerve paralysis after COVID-19 infection that led to pulmonary failure [17]. Compared to our patient, we believe the phrenic nerve affection led to the initial hiccups and then was complicated by paralysis, which resulted in pulmonary decompensation and intubation of the patient.

One common finding in patients presenting with persistent hiccups is having high inflammatory markers such as C-reactive protein (CRP) and acute kidney injury. Karakonstantis et al. reported a patient with lower lobe pneumonia presenting with persistent hiccups who then developed acute kidney injury preceded by high CRP [18]. Dorgalaleh et al. also reported a high rate of CRP in a patient with COVID-19 infection who developed prolonged hiccups [14]. Bakheet et al., in a patient with hiccups and COVID-19, found high rates of inflammatory markers, including CRP, ferritin, and lactate dehydrogenase (LDH) [9]. Similarly, our patient had signs of an inflammatory picture with high CRP, almost 10-fold the normal level, and developed acute kidney injury afterward.

Regarding investigational findings, several reports with chest infection have been linked in the literature with findings of ground-glass appearance on either chest x-rays or CT scans. Although our patient did not have hypoxia or signs of chest infection initially, he developed shortness of breath with no signs of pulmonary failure. Later, he started to develop signs of pneumonia, which presented with a ground-glass appearance on chest x-rays. All previous reports of hiccups had the same finding on either chest x-rays or CT scans [9,12,14]. Interestingly, our patient developed an anaphylactic reaction, which can be attributed to some allergic component that was largely in effect on the presentation of hiccups. Smokers have been linked to various allergic conditions, such as asthma, eosinophilic esophagitis, and rhinitis [19,20]. A case of eosinophilic esophagitis presenting with hiccups was reported in 2012 by Levy et al., who hypothesized that due to the irritation of the vagus nerve, the patient developed hiccups [21].

Many predisposing factors might lead to the development of hiccups in COVID-19 infection, such as a history of smoking, phrenic and vagus nerve damage or irritation, high inflammatory markers, and lower lobe pneumonia with a ground-glass appearance on x-rays. We hypothesize that hiccups are the first sign of serious deterioration of patients with COVID-19 and that such patients are at high risk of developing kidney injury and intubation.

## Conclusions

Patients with COVID-19 infection presenting with persistent hiccups and previous history of smoking should be monitored carefully. They have a higher chance of deterioration, causing serious complications such as acute kidney injury and pulmonary failure leading to intubation and anaphylactic shock.
